# Association Between Anaesthesiologists’ Sex and Anaesthesiology Quality Metrics and Postoperative Outcomes: A Retrospective Analysis

**DOI:** 10.3390/jcm13226986

**Published:** 2024-11-20

**Authors:** Sebastian Zeiner, Mathias Maleczek, Daniel Laxar, Razvan Bologheanu, Eva Schaden, Oliver Kimberger

**Affiliations:** 1 Clinical Division of General Anaesthesia and Intensive Care Medicine, Department of Anaesthesia, Intensive Care Medicine and Pain Medicine, Medical University of Vienna, 1090 Vienna, Austria; 2 Ludwig Boltzmann Institute Digital Health and Patient Safety, Medical University of Vienna, 1090 Vienna, Austria

**Keywords:** anaesthesia, perioperative care, quality assessment, sex

## Abstract

**Background:** Evidence suggests differences in medical practice and patient outcomes between male and female physicians and surgeons. To date, no such relationships were investigated in anaesthesiologists. This study aimed to investigate an association between anaesthesiologists’ sex and anaesthesia quality metrics as well as outcomes. **Methods:** We performed a population-based, single-centre, retrospective cohort study. Data were gathered from all patients undergoing anaesthesia between 1 January 2014 and 31 March 2022 at a large tertiary centre in Vienna, Austria. We examined 30-day mortality in relation to the sex of the anaesthesiologist after adjusting for various patient, physician, and hospital factors. Additionally, we assessed anaesthesiologists’ sex and several anaesthesia quality benchmarks. **Results:** The final dataset included 94,254 cases. The study showed a very small but statistically significant correlation between male anaesthesia providers and an elevated risk for all-cause mortality within 30 days (adjusted odds ratio [aOR]: 1.0026; 95% confidence interval [CI], 1.0003–1.0048). Both male and female anaesthesiologists demonstrated similar proficiency in managing hemodynamic stability, blood glucose levels, preventing postoperative acute kidney injury (AKI) and lung-protective ventilation. However, male anaesthesiologists showed slightly higher adherence to guidelines for PONV prophylaxis. **Conclusions:** In a dataset of nearly 95,000 cases, there was a clinically marginal but statistically significant association between male provider sex and 30-day mortality.

## 1. Introduction

Evidence suggests a difference in medical practice between male and female physicians concerning guideline adherence [1,2], preventive care [3,4] and communication skills [4,5]. Remarkably, elderly hospitalized patients treated by female physicians had lower mortality and readmission rates compared to those cared for by their male counterparts [6]. A recent study showed that patients had lower mortality and readmission rates when treated by female physicians. The effect was even larger for female patients treated by female physicians [7]. Similarly, patients treated by female surgeons had a reduced 30-day mortality compared to those treated by male surgeons [8]. This was confirmed in a recently published large cohort study that showed a significant increase in mortality in women treated by male surgeons [9]. While some studies on sex effects in surgical disciplines have been studied, data for anaesthesiology remains scarce. A study from 2009 claimed that anaesthesiologist sex has an effect on the mask ventilation learning process, with female residents finding it more difficult to provide a tight air seal in the early stage of training [10]. Wallis et al. examined the sex concordance of the surgeon–anaesthesiologist teams and found no association between sex discordance and overall patient outcome [9]. Considering these findings and a growing recognition of the influence the practitioner’s sex and gender may have on performance and overall outcomes, we aimed to investigate whether outcomes differ for patients cared for by female and male anaesthesiologists using a large, population-based cohort. Assessing sex-specific outcomes is important for tackling implicit bias and sex representations that might perpetuate existing inequalities [11,12].

As a first step towards understanding the effects anaesthesiologists’ sex might have, this study aimed to explore its association with outcomes after surgery as well as anaesthesia quality markers. Therefore, we performed a retrospective, single-centre, cohort study of patients undergoing surgery at the university-affiliated Vienna General Hospital. Anaesthesiologic performance metrics were assessed, including the incidence of postoperative AKI, occurrence of hemodynamic instability, use of PONV prophylaxis, glycaemic control, and lung-protective ventilation.

## 2. Materials and Methods

We conducted a retrospective cohort study including all patients undergoing surgery under any anaesthesia provided by anaesthesiologists at the Medical University of Vienna, Department of Anaesthesia, Intensive Care Medicine and Pain Medicine, Vienna, Austria between 1 January 2014 and 31 March 2022.

Data were extracted from the IntelliSpace Critical Care and Anaesthesia (ICCA; Philips GmbH Healthcare, Vienna, Austria) database and the Vienna General Hospital information management system (AKIM; Siemens AG Österreich, Vienna, Austria). After acquisition, patient data were anonymized, cleaned, and stored in a database. We excluded all cases longer than 12 h and cases with a handover/change of anaesthesiologist.

Ethical approval for this study (EK-Nr: 1304/2022) was provided by the Ethics Committee of the Medical University of Vienna, Austria (Chairperson Prof. Jürgen Zezula) on 3 June 2022.

### 2.1. Outcome Definition

The primary outcome was all-cause 30-day mortality and was collected by combining in-hospital mortality from electronic health records (EHR) with out-of-hospital mortality data obtained from the Austrian Federal Statisticians office.

Secondary outcomes included the following anaesthesia quality benchmarks:

Lung-protective ventilation: We calculated the percentage of cases where a low tidal volume of less than 8 mL/kg predicted BW was applied during general anaesthesia. At the patient level, the binary outcome is an indicator for the application of a low tidal volume of less than 8 mL/kg predicted BW.

Normothermia: We calculated the percentage of cases with normothermia, or at least one documented temperature >36° within 30 min before or 15 min after the anaesthesia end time. At the patient level, the binary outcome is an indicator of normothermia, or at least one documented temperature >36° within 30 min before or 15 min after the anaesthesia end time.

Haemodynamic Stability: We calculated the percentage of unique patient–provider instances where sustained intraoperative hypotension (<65 mmHg for at least 15 min) was avoided. At the patient level, the binary outcome is an indicator of avoiding sustained intraoperative hypotension (<65 mmHg for at least 15 min).

PONV prophylaxis: Apfel score was calculated and matched with the administration of antiemetics. At the patient level, the binary outcome is an indicator of adequate PONV prophylaxis.

Acute Kidney Injury (AKI): Postoperative creatinine was compared to preoperative values. AKI was defined as either a rise of 0.3 mg/dL within 48 h or 1.5mg/dL in seven days. If baseline creatinine values were missing, we calculated them based on the model developed by the Chronic Kidney Disease Epidemiology Collaboration [13].

Glycemic control: For this parameter, adequate reaction to either hyperglycemia (i.e., glucose level >200 mg/dL) or hypoglycemia (i.e., glucose level <60 mg/dL), namely the administration of insulin or glucose, respectively, and recheck was calculated. At the patient level, the binary outcome is an indicator of an adequate response in case of either hyperglycaemia (i.e., glucose level >200 mg/dL) or hypoglycaemia (i.e., glucose level <60 mg/dL).

### 2.2. Statistical Analysis

Continuous variables were summarized as means with standard deviations, and categorical variables were presented as absolute frequencies and percentages.

Python 3.8 (Python Software Foundation, Wilmington, NC, USA) with the packages pandas, numpy, scipy and statsmodels was used for all statistical calculations and modelling [14].

To estimate the relationship between anaesthesiologists’ sex and the primary outcome as well as secondary outcomes, we employed a linear mixed model. In this model, the following parameters were included in addition to the outcome in question: patient sex, age, body mass index, ASA score, duration of anaesthesia, duration of anaesthesia in concordance with ASA score, surgeon sex, Charlson morbidity score and number of previous cases with general anaesthesia by the provider.

To explore potential interactions between the procedure, patient, anesthesiologist, surgeon, and hospital characteristics, and the association between anesthesiologists’ sex and outcomes, subgroup analyses were conducted. We specifically examined the modification effect of patients’ sex, hypothesizing that female patients treated by male anaesthetists might have worse outcomes. Regarding procedural characteristics, we performed pre-planned stratified analyses based on the duration of surgery, ASA classification and Charlson Comorbidity score.

In this study, statistical significance was ascertained through a two-tailed comparison, with a determined *p*-value of less than 0.05.

## 3. Results

**Figure 1:**
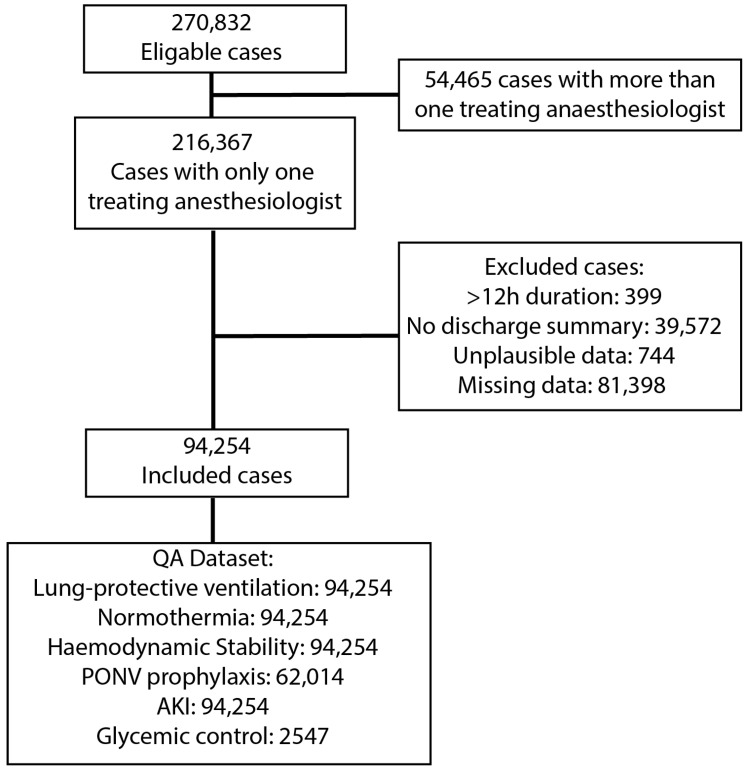
Patient flow chart. Figure 1 describes the cohort of patients included in the analyses.

We screened a total of 270,832 anaesthesia cases. After applying the exclusion criteria (Figure 1), the final dataset included 94,254 cases. Because of missing or unplausible data, 81,398 cases and 744 cases were excluded, respectively. For the quality benchmarks, the final case numbers are provided in the flow chart below (Figure 1).

Table 1 presents the baseline characteristics categorized by anaesthesiologists’ sex. Overall, 30-day mortality was low with 1.6% (n = 1473) of cases; 891 or 1.6% of those patients were cared for by male anaesthesiologists and 582 or 1.5% of those patients were cared for by female anaesthesiologists. However, female providers were more likely to care for sicker patients based on their ASA score, with 11,858 (30.1%) classified as ASA 3 and 1982 (5.0%) as ASA 4 compared to 15,818 (28.8%) and 2356 (4.3%) for male anaesthesiologists, respectively. Indeed, the Charlson Comorbidity score was higher for patients treated by female anaesthesiologists (0.9 (SD 1.5)) compared to those cared for by male anaesthesiologists (0.8 (SD 1.5)).

To better estimate the association between 30-day mortality and the sex of the anaesthesiologist, we corrected for patient sex and anaesthesia provider sex independently, along with various factors at the procedure, patient, anaesthetist, and hospital levels. The findings revealed a significant association between male anaesthesia providers and an increased likelihood of experiencing death of all causes within 30 days (adjusted odds ratio [aOR], 1.0026; 95% confidence interval [CI], 1.0003–1.0048). Further exploring the effects of sex concordance and discordance, we examined the mortality effects of male providers for female and male patients separately. This showed a higher effect on mortality for male patients treated by male anaesthesiologists (OR: 1.0033, CI: 1.0003–1.0064) compared to female patients (OR: 1.0019, CI: 0.9992–1.0047).

**Figure 2:**
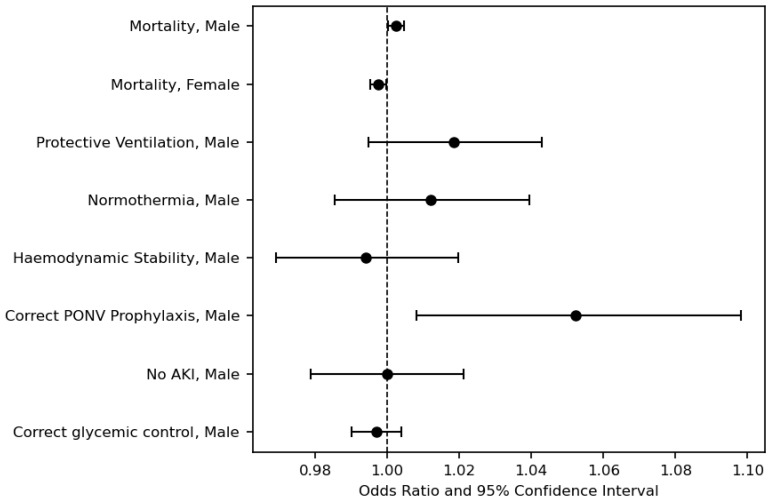
Adjusted odds ratios (95% CI). Figure 2 shows the adjusted odds ratios of all used quality parameters.

The performance concerning anaesthesia quality benchmarks by sex of the anaesthesiologist is provided in Table 2, and adjusted odds ratios are displayed in Figure 2. In our data, male anaesthesiologists demonstrated a slightly increased adherence to guidelines for providing adequate PONV prophylaxis (aOR: 1.0522; 95% CI 1.0080–1.0983). However, all other quality parameters did not differ significantly between the groups. The analysis of haemodynamic instability revealed no significant differences between male and female anaesthesiologists in avoiding sustained intraoperative hypotension (aOR: 0.9941; 95% CI 0.9691–1.0197). The percentage of unique patient–provider instances where sustained intraoperative hypotension (<65 mmHg for at least 15 min) was avoided was similar for male and female anaesthesiologists (61.7% and 60.5% of cases, respectively). The percentage of cases with an adequate response to hyperglycaemia (glucose level >200 mg/dL) or hypoglycaemia (glucose level <60 mg/dL), involving the administration of insulin or glucose, respectively, and recheck was comparable for both sexes (aOR: 0.9970; 95% CI 0.9901–1.0038). A low tidal volume of less than 8 mL/kg was applied similarly in both groups (32% and 27% for males and females, respectively, aOR 1.0186; 95% CI 0.9948–1.0043). The assessment of hypothermia management showed no significant disparities between male and female anaesthesiologists in maintaining normothermia or documenting appropriate temperatures within the specified timeframe (aOR 1.0121; 95% CI 0.9853–1.0395). Similarly, the proportion of cases with reduced renal function within the specified time window after anaesthesia was comparable for both sexes (aOR 0.9999; 95% CI 0.9789–1.0213).

## 4. Discussion

In this retrospective study conducted on a population-based cohort analysing anaesthesia quality and mortality, we found a significant—albeit only slightly—higher 30-day mortality in patients cared for by male anaesthesiologists after accounting for surgical speciality, patient and surgeon sex and several other patient factors. This finding is in line with current literature showing that physician sex plays a role in shaping patient outcomes, with female practitioners often being associated with better results [9,15,16]. While these factors are often neglected, these findings are consistent across various medical specialities and studies. In an observational study involving 1.1 million patients and close to 3000 surgeons in Canada, female surgeons were linked to a reduced risk of composite 30-day mortality, readmission, and morbidity compared to their male counterparts (female 11.1% vs. male 11.6%, adjusted odds ratio [aOR] 0.96, 95% confidence interval [CI]: 0.92–0.99) [8,9]. Moreover, an analysis of Medicare data covering over 1.5 million elderly patients admitted with common medical conditions revealed that patients treated by female physicians experienced lower 30-day mortality rates compared to those cared for by male physicians (females 11.1% vs. males 11.4%, aOR: −0.43, 95% CI: −0.57 to −0.28) [6]. Comparable results were observed among emergency physicians managing patients admitted with myocardial infarction [6].

The underlying factors for the observed disparity between practitioner sexes are unknown and most likely due to multifaceted and interlinked factors. Preceding research has proposed distinctions in communication styles, with women often dedicating more time to patient interaction and acquiring additional healthcare information, potentially influencing clinical care provision [17,18].

Effects of sex and gender on decision-making have been investigated outside of the healthcare sector as well. Research published in Judgment and Decision Making suggests gender disparities in risk perception and its influence on decision-making in risky situations [19]. The findings suggest that men and women may assess the probability and severity of potential negative outcomes differently. Specifically, women may demonstrate increased sensitivity to the likelihood of unfavourable outcomes or anticipate experiencing heightened emotional distress from such outcomes. Men typically exhibit riskier behaviour across multiple domains of life [19,20,21]. As an example, among US drivers, men are three times more likely than women to be involved in fatal car accidents; yet, it has to be taken into consideration that men are overall driving more miles than female drivers. [22]. Moreover, in alpine hiking, despite similar numbers of male and female hikers, women are less likely to die than men, with 3.5 times fewer female than male fatalities [23]. These differences in decision-making may transfer to clinical decision-making. For example, a 2017 study showed differences in the estimated risk of complications for lung resection by male and female surgeons [24]. The same researchers also showed that male and female surgeons perceived frailty differently when assessing videos of patients as well as associated surgical recommendations [25].

We aimed to further explore differences in decision-making and perception by closely examining several anaesthesia quality markers and the associated adherence to guidelines. Quality markers assessed were haemodynamic instability, PONV prophylaxis, glycaemic control, lung-protective ventilation, hypothermia, and AKI management.

Both sexes demonstrated comparable proficiency in managing haemodynamic stability during procedures, with no statistically significant disparities observed.

Furthermore, male and female anaesthesiologists demonstrated similar effectiveness in responding to abnormal blood glucose levels, ensuring adequate glycaemic control for their patients. Additionally, male and female anaesthesiologists exhibited comparable adherence to lung-protective ventilation practices, with no sex-related variations in the application of ventilation strategy. Likewise, both male and female anaesthesiologists were equally successful in managing patients’ body temperatures during the perioperative period, with no substantial differences observed.

Our results suggest that male anaesthesiologists exhibited an increased adherence to PONV guidelines compared to female anaesthesiologists. This contradicts previous literature that showed higher guideline adherence by female physicians for heart failure [2]. PONV, however, predominantly affects women and this might be a contributing factor. A study on differences in recommended treatment by male and female physicians showed more guideline adherence in drug recommendations and higher target doses in patients treated by female physicians [1]. No different treatment for male or female patients by female physicians was observed while male physicians used significantly less medication and lower doses in female patients [1]. Guideline adherence could contribute to enhanced quality of care and improved patient outcomes [2]. It is important to note that adherence to PONV was the only quality marker showing a difference between male and female providers. This shows that the female providers were, in general, not less adherent to guidelines. Future research will have to analyse this specific finding.

### Limitations

To the best of our knowledge, this study is the first to investigate the relationship between anaesthesiologists’ sex, anaesthesia quality, and patient outcomes. Nevertheless, given the monocentric observational design of this study, it is important to acknowledge its limitations. Our data collection relied on self-reported biological sex, thus precluding the evaluation of gender as well as other social identities. Furthermore, we cannot include or independently assess the influence of other members of the team, such as nurses and students, due to the absence of this information in our database. Further research will need to confirm the generalizability of our results across multiple centres.

Another possible source of bias is the immediacy of our data. In this study, data up until 2022 was included as the federal statistician’s office only provides mortality data with a specific delay. Therefore, no data for 2023 was available at the time of model calculation.

Dataset building plays an important role in introducing possible risks of bias. As shown in Figure 1, a large proportion of cases had to be excluded either due to missing data or due to missing patient summaries. The missing patient summaries most often occurred in the process of merging our two institutional patient databases. Cases with missing values like weight or height had to be excluded to enable the pre-specified analysis. In the eligible cases, 42.1% of providers were female; the dataset with only missing data contained 42.3% females, resulting in 41.8% female providers in the final dataset. This shows that the distribution of gender did not change due to the application of exclusion criteria. A small sensitivity analysis showed that applying exclusion criteria excluded a greater proportion of ASA 1 cases and younger patients. This is likely due to the decreased quality of documentation in those usually shorter cases. The distribution of cases between genders did not change.

The large variety of surgeries and surgeons in the presented cohort may introduce bias. In order to enhance the generalizability of the results, it was decided not to limit the cohort. However, the law of large numbers should compensate for these varieties in such a large dataset.

It is crucial to highlight that the results presented only indicate associations and do not establish any causal relationships. Therefore, we cannot draw definitive conclusions about the underlying reasons for the observed differences.

## 5. Conclusions

In conclusion, this retrospective study found a marginally higher 30-day mortality among patients cared for by male anaesthesiologists, taking into consideration the variation in patient, physician, and hospital factors. Both male and female anaesthesiologists demonstrated similar proficiency in managing hemodynamic stability, blood glucose levels, avoiding AKI and using lung-protective ventilation. However, male anaesthesiologists showed slightly higher adherence to guidelines for PONV prophylaxis. Further investigation into the drivers behind these observations among practitioners is needed.

## Figures and Tables

**Table 1:** Baseline characteristics of the study cohort, stratified by anaesthesia provider’s sex. Chi2 and *t*-tests were used to calculate unadjusted *p*-values. Note that *p* values tend to be low in large numbers of observations. BMI: body mass index.

	Overall	Male Provider	Female Provider	*p*
Age (SD)	49.8 (21.9)	49.5 (22.1)	50.2 (21.7)	*p* < 0.001
Weight (SD)	74.2 (23.4)	74.2 (23.9)	74.2 (22.5)	0.95
BMI (SD)	26.1 (6.5)	26.1 (6.6)	26.0 (6.2)	0.2
Duration of surgery [h]	2.4 (1.7)	2.4 (1.7)	2.3 (1.7)	*p* < 0.05
Sex concordance patient/provider (%)	47,309 (50.2%)	26,383 (48.1%)	20,926 (53.1%)	*p* < 0.001
Male patient gender (%)	44,838 (47.6%)	26,383 (48.1%)	18,455 (46.9%)	*p* < 0.001
Female patient gender (%)	49,357 (52.4%)	28,431 (51.9%)	20,926 (53.1%)	*p* < 0.001
Unknown patient gender (%)	59 (0.1%)	26 (0.0%)	33 (0.1%)	*p* < 0.001
Male surgeon (%)	70,663 (75.0%)	41,402 (75.5%)	29,261 (74.2%)	*p* < 0.001
Female surgeon (%)	23,591 (25.0%)	13,438 (24.5%)	10,153 (25.8%)	*p* < 0.001
ASA classification: n (%)				*p* < 0.001
ASA 1	24,732 (26.2%)	14,614 (26.6%)	10,118 (25.7%)	-
ASA 2	36,779 (39.0%)	21,624 (39.4%)	15,155 (38.5%)	-
ASA 3	27,676 (29.4%)	15,818 (28.8%)	11,858 (30.1%)	-
ASA 4	4338 (4.6%)	2356 (4.3%)	1982 (5.0%)	-
ASA 5	725 (0.8%)	425 (0.8%)	300 (0.8%)	-
Charlson Comorbidity score (SD)	0.8 (1.5)	0.8 (1.5)	0.9 (1.5)	0.43
LOS (SD) [days]	14.2 (29.5)	14.2 (29.5)	14.2 (29.6)	0.98
30-day mortality	1473 (1.6%)	891 (1.6%)	582 (1.5%)	0.07
Surgical area, n (%)				*p* < 0.001
Urology, gynaecology, general surgery	16,968 (18.0%)	10,383 (18.9%)	6585 (16.7%)	-
Maxillofacial, ENT, derma	12,327 (13.1%)	7177 (13.1%)	5150 (13.1%)	-
Neurosurgery	4871 (5.2%)	2833 (5.2%)	2038 (5.2%)	-
Non-OR anaesthesia, obstetrics	6888 (7.3%)	3849 (7.0%)	3039 (7.7%)	-
Robotic surgery	1689 (1.8%)	1027 (1.9%)	662 (1.7%)	-
Cardiothoracic and vascular surgery	9741 (10.3%)	4963 (9.0%)	4778 (12.1%)	-
Orthopaedics, trauma	18,949 (20.1%)	11,489 (21.0%)	7460 (18.9%)	-

**Table 2:** Anaesthesia quality benchmarks by anaesthesiologist sex. Counts (n) and percentage (%) of cases per sex. AKI: acute kidney injury, OR: operation, PONV: post operative nausea and vomitting.

Benchmark	Male (n,%)		Female (n,%)	
normothermic	28,868	52.64%	19,473	49.41%
no AKI post OR	31,705	91.28%	23,062	91.81%
normotension	33,830	61.69%	23,852	60.52%
protective ventilation	14,330	31.56%	8682	27.48%
correct glycaemic control	1398	99.15%	1131	99.47%
PONV prophylaxis	16,394	45.53%	10,385	39.94%

## Data Availability

The data presented in this study are available on request from the corresponding author due to privacy/legal restrictions.
